# Ethanol Extract of Root of* Prunus persica* Inhibited the Growth of Liver Cancer Cell HepG2 by Inducing Cell Cycle Arrest and Migration Suppression

**DOI:** 10.1155/2017/8231936

**Published:** 2017-10-12

**Authors:** Hongchun Shen, Honglian Wang, Li Wang, Lu Wang, Menglian Zhu, Yao Ming, Sha Zhao, Junming Fan, En Yin Lai

**Affiliations:** ^1^College of Integrated Chinese and Western Medicine, Southwest Medical University, Luzhou, Sichuan 646000, China; ^2^Laboratory of Organ Fibrosis Prophylaxis and Treatment by Combine Traditional Chinese and Western Medicine, Research Center of Combine Traditional Chinese and Western Medicine, Affiliated Traditional Medicine Hospital of Southwest Medical University, Luzhou, Sichuan 646000, China; ^3^Department of Nephrology, The Affiliated Hospital of Southwest Medical University, Luzhou, Sichuan 646000, China; ^4^Department of Nephrology, The Affiliated Traditional Medicine Hospital of Southwest Medical University, Luzhou, Sichuan 646000, China; ^5^Chengdu Medical College, Chengdu, Sichuan 610041, China; ^6^Department of Physiology, Zhejiang University School of Medicine, Hangzhou 310000, China

## Abstract

Liver cancer is the second most lethal cancer and hepatocellular carcinoma (HCC) is the primary cancer subgroup. However, the current chemotherapy agents remain ineffective and present wide side effects for advanced HCC patient. In this study, we investigated the antitumor role of ethanol extract of root of peach tree (*Prunus persica (L.) Batsch *and hereafter designated as TSG in short of its Chinese name), which is an important ingredient in Chinese medicine prescription, in liver cancer cell HepG2. By cell viability assay, we showed that addition of TSG in the culture medium inhibited the cell growth of HepG2 cells in a dose and time-dependent way. Cell cycle analysis indicated that TSG caused sustained M/G2 phase arrest. The expression of mitosis-related protein Cdc25c was impaired upon TSG treatment. Furthermore, wound healing assay demonstrated that TSG treatment notably suppressed the migration of HepG2 cells and the expression of extracellular matrix metalloprotease, MMP3 and MMP9. Most significantly, administration of TSG inhibited* in vivo* tumor growth in nude mice. Our findings suggested that TSG may serve as a source to isolate anti-HCC therapeutic ingredients.

## 1. Introduction

According to the epidemiological data from the world health organization, Liver cancer is the second most lethal cancer type around the world. And, hepatocellular carcinoma (HCC) is the most common primary liver cancer [1]. Although early-staged HCC can be subjected to curative surgical treatments, like tumor resection and ablation, the majority of HCC patients often already progress to intermediate or advanced disease stage upon clinical diagnosis. In such case, palliative treatment is the primary medical choice. However, even the first line palliative treatment medicine, sorafenib, contributes modest survival benefit (about 2.5 months) to advanced HCC patient [2, 3]. Thus, there is an urgent demand for developing novel therapeutic drugs for future HCC therapy.

*Prunus persica *(L.) Batsch, peach tree or taoshu in Chinese, originated from Northwest China and nowadays is a worldwide cultivated fruit plant [4]. Beyond usage as a food source, the physical parts of peach tree also showed biological activity and contained constituents with medical effects. Like other fruits, the juicy flesh of peach contains various kinds of secondary metabolites with antioxidant and anti-inflammation activity [5]. In addition, physical parts of* Prunus persica* are used as important ingredients in traditional Chinese medicine prescriptions.* Prunus persica* has been included in the traditional Chinese medicine prescription to treat blood stasis [6]. Fukuda et al. reported that several isolated glycosides from* Prunus persica* seeds can inhibit* in vitro *and* in vivo* tumorigenesis [7]. Recently, it is shown that the polyphenolic extract of* Prunus persica* fruit can suppress breast cancer cell proliferation and tumor growth [8, 9]. Furthermore, bark extract of* Prunus africana*, also named* Pygeum africanum* and a species showing evolutionarily close relationship with* Prunus persica*, has been successfully used in the treatment of benign prostatic hyperplasia [10, 11].

Root of* Prunus persica* serves as another important ingredient in traditional Chinese medicine prescriptions. It is described to have the function to treat disease like jaundice, hematemesis, hemorrhoids, amenorrhea, and tinea in classical Chinese medical documents and also used in nowadays Chinese medicine application. The preliminary pharmacological study indicated that root of* Prunus persica* have anti-inflammation activity [12]. In experience-based clinical application, root of* Prunus persica* was also applied to treat esophageal cancer [13]. In this study, we firstly investigated the antitumor effect of ethanol extract of root of* Prunus persica* on the hepatocarcinoma cell HepG2. Our results indicated that the ethanol extract of root of* Prunus persica* can impair HepG2 cell viability and suppress cell growth through cell cycle arrest and inhibit cell migration. Importantly, administration of ethanol extract of root of* Prunus persica* can efficiently suppress the tumor growth in nude mice.

## 2. Materials and Methods

### 2.1. Drug and Cell Line

The dry root of* Prunus persica* (L.) Batsch, also taoshugen (TSG) in its Chinese name, was subjected to mechanical comminution followed by ethanol extraction for 48 hours at room temperature. The insoluble particles were removed by centrifugation and filtration through a 0.22 *μ*m membrane. The bulk ethanol extract was vacuum dried and quantified. For* in vitro* cellular assay, DMSO was used to redissolve the dried ethanol extract, which can be fully dissolved in DMSO with a maximum solubility of about 0.12 g/ml. For animal experiment, the dried extract was further redissolved in ethanol and intragastrically administrated to the nude mice at indicated concentration in 10% ethanol. HepG2 cell line was a kind gift from Professor Qin Zhou from Chongqing Medical University and was cultured in DMEM supplemented with 10% FBS (Gemini, USA) at 37°C with 100% humidity and 5% CO_2_.

### 2.2. Cell Viability Assay

Total cell viability was determined with CellTiter 96@ AaQueous One Solution (promega, USA). HepG2 cells were seeded on 96-well plate at a density of 3000 cells per well. On the next day, medium containing drugs of indicated concentration or equivalent DMSO was added. 24 hours later, medium was replaced with fresh growth medium containing 10% of the above cell viability assay reagent and incubated for 1 hour. The plate was analyzed with BioTek synergy 2 microplate reader (USA). All treatments were performed in 4 replicates on the same plate.

### 2.3. Cell Cycle Analysis

Cell cycle analysis was conducted with the cell cycle detection kit (KeyGen, China) according to the manufacturer's instruction. Briefly, cells subjected to indicated treatment were harvested by regular trypsin digestion and rinsed with PBS. After fixation in 70% ethanol overnight at −20°C, cells were rinsed again in PBS and stained in staining solution containing PI and RNase A. Flow cytometry (Canto II, BD bioscience, USA) was employed to analyze the cellular DNA content. Each treatment was performed in triplicate.

### 2.4. Western Blotting

For western blotting, 1 × 10^6^ cells were seeded onto 6 cm dish. After subjecting to indicated treatment for 24 hours, the cells were detached with trypsin digestion and rinsed with PBS followed by lysis with RIPA buffer and centrifugation to remove debris. Protein concentration was determined with the coomassie brilliant blue method. Western blotting was performed as previously described [14]. Antibodies used in this study included rabbit anti-Cdc25c polyclonal antibody (Cat# D154112, Sangon Bio-tech, China), rabbit anti-CDK1 polyclonal antibody (Cat# D160158, Sangon Bio-tech, China), rabbit anti-MMP9 polyclonal antibody (Cat# 10375-2-AP, Proteintech, USA), rabbit anti-MMP3 polyclonal antibody (Cat# 17873-1-AP, Proteintech, USA), mouse anti-*β*-Actin monoclonal antibody (Cat# TA-09, ZSGB-Bio, China), HRP-conjugated goat anti-mouse polyclonal antibody (Cat# ZB2305, ZSGB-Bio, China), and HRP-conjugated goat anti-rabbit polyclonal antibody (Cat# ZB2301, ZSGB-Bio, China).

### 2.5. Wound Healing Assay

HepG2 cells were seeded onto 6-well plate at a density of 2 × 10^6^ per well. Next day, uniform scratch was made with a pipette tip on the culture surface with 100% cell confluence. The well was rinsed with fresh blank DMEM medium two times and the cells were maintained in another 2 ml of blank DMEM medium or DMEM medium with 0.34 mg/ml TSG. Cell migration status was monitored at indicated time point under microscope and represented by the recovery of the scratch.

### 2.6. *In Vivo* Animal Experiment

The athymic BALB/c nude mice (BALB/cJNju-Foxn1nu/Nju, 2-month old with body weight of about 17 g, Dashuo Biotech) were subcutaneously inoculated with 1 × 10^6^ HepG2 cells on both sides of the posterior back, respectively. Since the 2nd day after cancer cell inoculation, the mice were intragastrically administrated with 4.3 mg/day of TSG or equivalent volume of solvent (10% ethanol) for 4 weeks. Each group contains 10 mice. The concentration of the stock ethanol extract of TSG is 0.086 g/ml which almost reaches saturation. In consideration of a maximum administration dosage, the maximum volume tolerance of intragastrical drug administration and avoiding hazards from high concentration of ethanol, the stock TSG ethanol extract was diluted 10-fold with distilled water (finally in 10% ethanol) and intragastrically administrated to nude mice in 0.5 ml/day (with a final dosage of 4.3 mg/day). At the end of drug treatment, the mice were anaesthetized by pentobarbital sodium and the tumors were harvested followed by photography and weighing. The animal experiment was approved by the animal ethics committee of Southwest Medical University and conformed to corresponding regulations.

## 3. Results

### 3.1. TSG Induced HepG2 Cell Growth Inhibition

**Figure 1:**
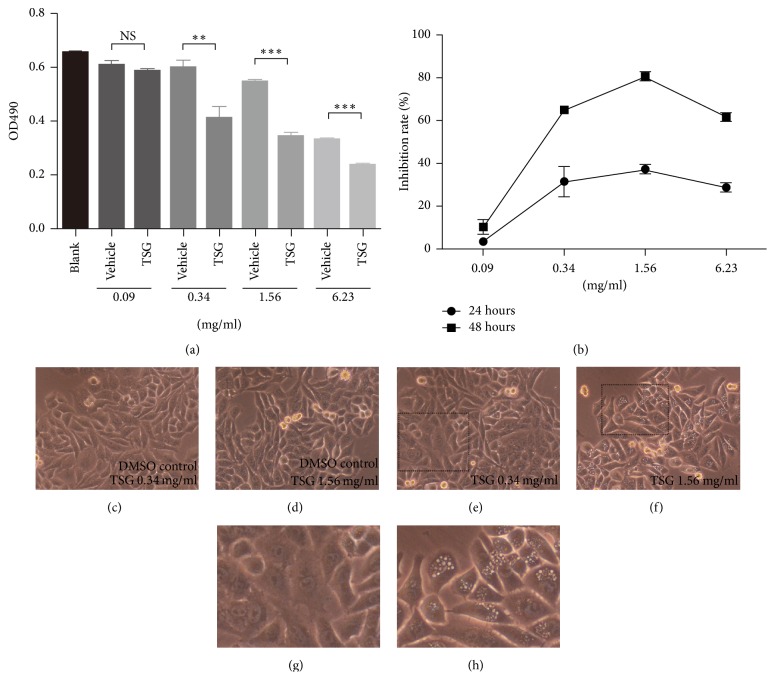
TSG inhibited growth of HepG2 cells. (a) The HepG2 cells were treated with TSG of various concentration for 24 hours followed by determination of total cell viability by viability assay. ^*∗∗*^*p* < 0.01. ^*∗∗∗*^*p* < 0.001. NS: no statistical significance. (b) The dosage and time-dependent growth inhibition rate was calculated based on data from viability assay. ((c)–(h)) Morphological change of HepG2 cells treated with TSG and corresponding vehicle control for 24 hours. (g) and (h) are enlarged image in (e) and (f), respectively.

To begin the anti-HCC activity investigation of root of* Prunus persica*, we firstly performed the ethanol extraction of root of* Prunus persica *(TSG thereafter). For cell experiment, the solvent ethanol was replaced with DMSO as described in the method section. To test the effect of TSG on the growth of liver cancer cell HepG2, we treated the cells with TSG of different concentration and the cell viability was monitored at 24 and 48 hours. The relative growth inhibition rate was calculated with the corresponding vehicle (DMSO) group as control since DMSO were observed to demonstrate slight toxicity on HepG2 cells when used at higher concentration. As shown in Figure 1, compared to vehicle group, TSG treatment induced time and dosage-dependent growth inhibition with a decreased total cell viability. 1.56 mg/ml TSG treatment for 24 hours can cause 37% growth inhibition (Figures 1(a) and 1(b)). Morphologically, TSG treatment caused cytoplasmic vacuolization which also demonstrated a dosage effect (Figures 1(c)–1(h)).

### 3.2. TSG Treatment Caused Cell Cycle Arrest in G2/M Phase

**Figure 2:**
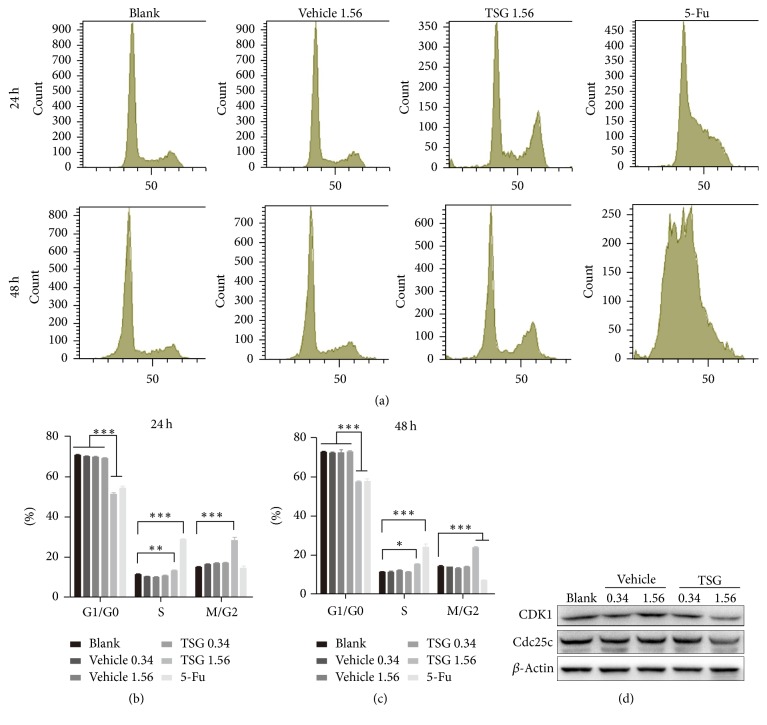
TSG treatment induced G2/M phase arrest of HepG2 cells. (a) Flow cytometry analysis of DNA content of HepG2 cells subjected to indicated treatment. ((b), (c)) Statistical analysis of cell subpopulation ratios for each phase of cell cycle. ^*∗*^*p* < 0.05. ^*∗∗*^*p* < 0.01. ^*∗∗∗*^*p* < 0.001. (d) Western blotting analysis to detect the expression of CDK1 and Cdc25c.

To investigate the factors contributing to the inhibited growth of HepG2 cells, we checked the effect of TSG on cell cycle. As revealed by PI staining followed by flow cytometry analysis, high-dosage of TSG (1.56 mg/ml) significantly disturbed the cell cycle with increased M/G2 subgroup and decreased G1/G0 subgroup (*p* < 0.001). And this cell cycle arresting effect can last for at least 2 days. However, treatment with DMSO and low-dosage TSG (0.34 mg/ml) had no influence on cell cycle (Figures 2(a)–2(c)). As a control, 5-fluorouracil (5-Fu, 5 *μ*g/ml) also demonstrated vigorous disturbance of HepG2 cell cycle which keeps in line with the previous report [15]. To unravel the underlying molecular event associated with the altered cell cycle pattern, western blotting was used to analyze the key factors regulating cell cycle progress. As shown in Figure 2(d), the protein expression level of Cdc25c and CDK1, the key proteins modulating mitosis-phase entry, decreased upon treatment with high dose of TSG. In line with the DNA content analysis, low dose of TSG and DMSO (vehicle) showed no influence on protein level of Cdc25c and CKD1. These findings suggested that TSG induced cell cycle arrest at proper concentration.

### 3.3. The Migration Ability of HepG2 Cells Was Impaired by TSG

**Figure 3:**
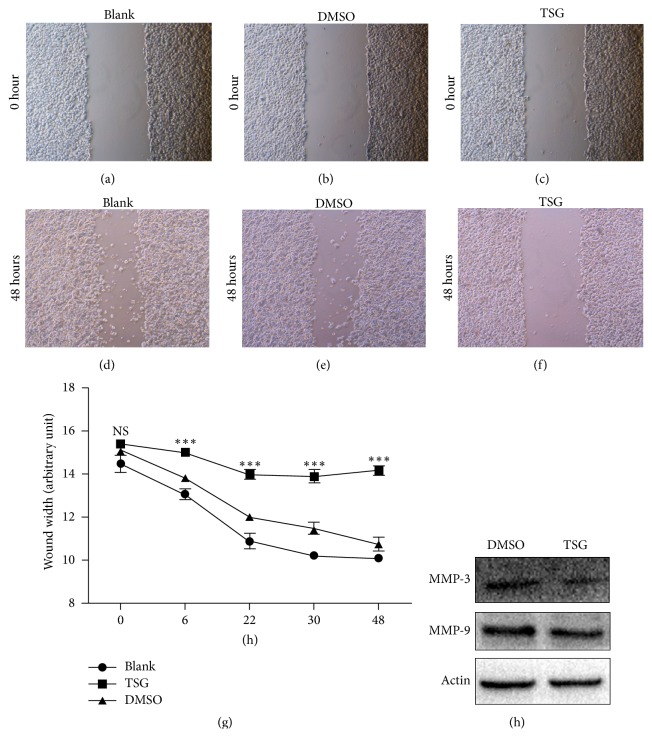
TSG suppressed HepG2 cell migration. ((a)–(f)) Representative images of wound healing assay of HepG2 cells treated with 0.34 mg/ml TSG or equal volume of DMSO or blank medium at the time point of 0 and 48 hours. (g) Statistical analysis of wound width of HepG2 cells with indicated treatment at indicated time point. ^*∗∗∗*^*p* < 0.001 versus TSG and DMSO group. (h) Western blotting analysis of protein level of MMP-3 and MMP-9.

As increased migration is another important feature of cancer cell, we then turned to investigate the influence of TSG on migration ability of HepG2 cells. The wound healing assay was employed to analyze the migration ability of HepG2 cells treated with TSG in basal medium without serum. As higher concentration of TSG (1.56 mg/ml) caused severe cell death in serum-free condition, the lower TSG dose (0.34 mg/ml) was used in this assay (data not shown). As shown in Figures 3(a) and 3(b), TSG-treated cells demonstrated notable compromised migration since early 6 hours after drug administration. This impaired migration was even more obvious with longer time of TSG treatment. In contrast, the addition of DMSO had no effects on cellular migration compared with the control (blank) (Figures 3(a)–3(g)). Accordingly, TSG treatment attenuated the expression level of the migration marker protein MMP3 and MMP9 as revealed by western blotting (Figure 3(h)).

### 3.4. TSG Inhibited* In Vivo* Tumor Growth in Nude Mice

**Figure 4:**
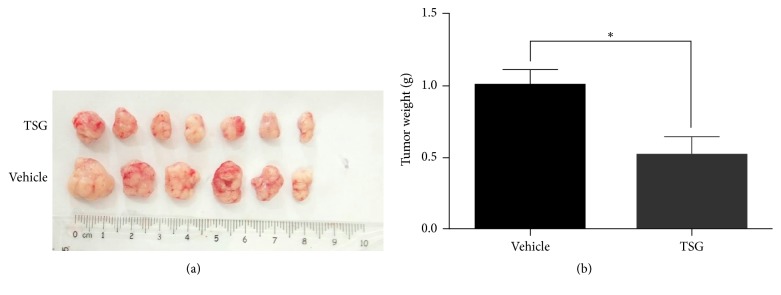
TSG attenuated HepG2-derived* in vivo* tumor growth. (a) Representative image of isolated tumors from tumor-bearing nude mice treated with TSG or solvent solution. (b) Statistical analysis of weight of isolated tumors. ^*∗*^*p* < 0.05 versus control group.

To unravel whether TSG can present a suppression activity on* in vivo* tumor growth, we established the live tumor model by subcutaneously transplanting HepG2 cells in nude mice which were subjected to intragastrical administration of TSG (4.3 mg/day) or vehicle solution. One month later, the TSG-treated nude mice developed smaller tumors in respect to both size and weight compared with the vehicle group (Figures 4(a) and 4(b)). These results implicated that TSG can partially inhibit* in vivo* tumor growth derived from HepG2 cells.

## 4. Discussion

*Prunus persica *is an important source of medicinal materials in traditional Chinese medicine. Physical parts of* Prunus persica*, like flower, seed, and gum, have been used to cure disease for hundreds of years. Root of* Prunus persica* is applied to treat jaundice, hematemesis, hemorrhoids, amenorrhea, and tinea in Chinese medicine. In experience-based folk medicine, root of* Prunus persica* is also included in the prescription to ameliorate esophageal cancer [13]. As far as we know, only one study described the toxicity of root of* Prunus persica* on liver cancer cell lines SMMC7721 and SK-HEP-1 [13]. However, no detailed mechanism on its antitumor effect was provided. In this study, we presented the evidence that ethanol extract of root of* Prunus persica* can inhibit the growth of HepG2 cells* in vitro* by impairing cell cycle and migration. Most importantly, ethanol extract of root of* Prunus persica* can slow down the* in vivo* growth of HepG2 cells-derived tumor in nude mice.

Uncontrolled cell proliferation, apoptosis, cell cycle, and enhanced cell migration are the key features of tumor cells and also the important therapeutic targets. In line with the previous report in liver cancer cell SMMC7721 and SK-HEP-1, our data indicated that root of* Prunus persica* extract also induced a dose and time-dependent growth inhibition of HepG2 cells. Furthermore, cell cycle analysis demonstrated a sustained M/G2 phase arrest. However, we found that the apoptosis of HepG2 cells showed little change upon treatment with root of* Prunus persica* extract at the concentration tested (data not shown). On the other hand, migration of HepG2 cells was significantly impeded by root of* Prunus persica* extract companioned with decreased expression of extracellular matrix metalloprotease, MMP3 and MMP9. These data implied that root of* Prunus persica* extract-induced HepG2 growth inhibition may be attributed to cell cycle arrest and migration suppression.

Furthermore, it is recently reported that the polyphenolic extract of* Prunus persica* fruit can inhibit the proliferation of* in vitro* breast cancer cell and* in vivo* breast tumor growth and lung metastasis [8, 9, 16]. The* Prunus persica* fruit and its polyphenolic extract can also execute chemoprotective role against cancer through modulating* in vivo* xenobiotic metabolizing enzymes [17]. Fukuda et al. reported that* Prunus persica *seed-derived glycosides showed antitumor activity* in vitro* and* in vivo* [7]. Combining these studies with our findings, we may speculate that many physical parts of* Prunus persica *tree contain antitumor components. However, more studies are needed to confirm this hypothesis.

By now, chemotherapeutic palliative treatment is almost the only choice for advanced liver HCC. However, molecular targeting agents, like multitarget kinase inhibitor sorafenib and sunitinib, contribute limited survival benefit to HCC patients [3, 18]. And cytotoxic agents, like cisplatin and doxorubicin, show almost no therapeutic response [3]. Most importantly, both of the molecular targeting agents and cytotoxic agents demonstrate severe side effects [3]. On the contrary, root of* Prunus persica* is a natural material and used as medical ingredient for hundreds of years in Chinese medicine. The long time clinical application suggests its relative safety. Thus, it should be a good source to explore novel HCC chemotherapy agent. However, strictly controlled study is needed to assess the safe dosage and potential side effects before its translation to clinical application. On the other hand, we should note that the ethanol extract of root of* Prunus persica* used in this study is a complex mixture may containing several different therapy-effective components and also impurities. This makes deep exploration of the tumor suppression role of root of* Prunus persica* difficult. Thus, our next work is to isolate and identify these medical active ingredients to further clarify their anti-HCC mechanism.

## 5. Conclusion

Ethanol extract of root of* Prunus persica* can inhibit* in vitro* HepG2 cells growth by inducing cell cycle arrest and migration suppression and* in vivo* tumor growth, suggesting potential application for novel anti-HCC drug development.
